# Coping Mechanisms, Psychological Distress, and Quality of Life Prior to Cancer Genetic Counseling

**DOI:** 10.3389/fpsyg.2018.01218

**Published:** 2018-07-16

**Authors:** Valentina E. Di Mattei, Letizia Carnelli, Martina Bernardi, Rebecca Bienati, Chiara Brombin, Federica Cugnata, Emanuela Rabaiotti, Milvia Zambetti, Lucio Sarno, Massimo Candiani, Oreste Gentilini

**Affiliations:** ^1^Faculty of Psychology, Vita-Salute San Raffaele University, Milan, Italy; ^2^Clinical and Health Psychology Unit, Department of Clinical Neurosciences, IRCCS San Raffaele Hospital, Milan, Italy; ^3^Department of Psychology, University of Milano-Bicocca, Milan, Italy; ^4^Language Abilities Department, University of Parma, Parma, Italy; ^5^University Centre of Statistics in the Biomedical Sciences, Vita-Salute San Raffaele University, Milan, Italy; ^6^Department of Obstetrics and Gynecology, IRCCS San Raffaele Hospital, Milan, Italy; ^7^Department of Medical Oncology, IRCCS San Raffaele Hospital, Milan, Italy; ^8^Faculty of Medicine, Vita-Salute San Raffaele University, Milan, Italy; ^9^Breast Surgery and Breast Unit, IRCCS San Raffaele Hospital, Milan, Italy

**Keywords:** cancer genetic counseling, psychological distress, coping mechanisms, quality of life, pre-test distress

## Abstract

**Background:** Breast Cancer susceptibility genes 1 and 2 are implicated in hereditary breast and ovarian cancer and women can test for the presence of these genes prior to developing cancer. The goal of this study is to examine psychological distress, quality of life, and active coping mechanisms in a sample of women during the pre-test stage of the genetic counseling process, considering that pre-test distress can be an indicator of post-test distress. We also wanted to identify if subgroups of women, defined based on their health status, were more vulnerable to developing distress during the genetic counseling process.

**Methods:** This study included 181 female participants who accessed a Cancer Genetic Counseling Clinic. The participants were subdivided into three groups on the basis of the presence of a cancer diagnosis: Affected patients, Ex-patients, and Unaffected participants. Following a self-report questionnaire, a battery of tests was administered to examine psychological symptomatology, quality of life, and coping mechanisms.

**Results:** The results confirm that the genetic counseling procedure is not a source of psychological distress. Certain participants were identified as being more vulnerable than others; in the pre-test phase, they reported on average higher levels of distress and lower quality of life. These participants were predominantly Ex-patients and Affected patients, who may be at risk of distress during the counseling process.

**Conclusions:** These findings highlight that individuals who take part in the genetic counseling process are not all the same regarding pre-test psychological distress. Attention should be paid particularly to Ex-patients and Affected patients by the multidisciplinary treating team.

## Introduction

According to the Italian Register of Cancer, in 2016 in Italy 50,000 and 5,200 women received a diagnosis of breast and ovarian cancer, respectively (AIOM^1^; Associazione Italiana Registri Tumori and Associazione Italiana di Oncologia Medica (AIOM and AIRTUM), 2016). Between 5 and 7% of the breast cancer cases are due to hereditary factors, and 25% of these are down to genetic mutations in BRCA1 and BRCA2 genes. Advances in molecular genetics have led to the cloning of these two breast-ovarian cancer susceptibility genes, which are inherited in an autosomal dominant Mendelian manner (Pasacreta, 1999). In women who carry the BRCA1 mutation the chances of getting breast cancer are around 65% and with BRCA2 they are around 40% (AIOM). Focusing on ovarian cancer, around 5–10% of the total cases are due to familial or hereditary forms of the disease. The BRCA1 gene is mutated in around 5% of cases of women under 70 years of age, while the presence of either BRCA1/2 in women over 70 is around 20–60% (Associazione Italiana Registri Tumori and Associazione Italiana di Oncologia Medica (AIOM and AIRTUM), 2016). Since these two types of cancer, especially breast cancer, are so prevalent in the population it is important to identify the genes that increase the risk of hereditary breast and ovarian cancer (HBOC) early on during the diagnostic process. Fortunately, genetic testing techniques have come a long way and there is a possibility of identifying the genes responsible for HBOC even before a cancer diagnosis has been made.

Women who have been identified as genetically at risk for developing HBOC have the choice of several interventions: surveillance, prophylactic mastectomy, prophylactic salpingo-oophorectomy, and chemoprevention via, for instance, the use of tamoxifen (Burke et al., 1997). Risk-reducing oophorectomy in women with either BRCA1/2 mutations reduces ovarian cancer mortality and breast cancer risk (Kauff et al., 2008; Domchek et al., 2010). Moreover, risk-reducing mastectomy is also effective in reducing the chance of developing breast cancer (Hartmann et al., 1999). Each of these prophylactic interventions has its limitations and disadvantages (Grann et al., 1998; Goodwin, 2000; Pasacreta et al., 2002) for instance, elective oophorectomy has been implicated in early menopausal symptoms (Finch et al., 2011). However, by undertaking these preventative strategies, women have the chance to reduce their risk of developing cancer in the future.

HBOC testing is usually carried out in a clinical setting as part of an all-inclusive program that consists of pre- and post-test counseling, treatment options, habitual follow-up appointments, and monitoring. It is crucial to comprehend the behavioral and psychological effects of providing information about genetic risk (Buckmaster and Gallagher, 2010). The American Society of Clinical Oncology (2015) upholds that genetic testing should be made available to individuals with suspected inherited cancer risk, where test results can be understood and when these can influence the medical management of the patient.

Genetic counseling is an intervention aimed at informing and supporting individuals who must undertake a medical path that searches for and manages genetic defects or pathologies (NHS Choices, 2016). Usually the genetic counseling process and testing procedure for HBOC takes the following form (Kash and Lerman, 1998): (A) *Selection:* if a woman takes part in genetic testing it is usually down to a referral from their general practitioner or their oncologist. These experts may feel it necessary for a woman to undergo testing because there are signs that there may be a familial link to either breast or ovarian cancer. Thus, a multidisciplinary team (composed of an oncologist, geneticist, and genetic counselor) evaluate each individual case and decide whether a patient should be subjected to the genetic testing procedure. (B) *Pre-testing stage:* during this stage, the individual learns about all the familial, hereditary, and sporadic forms of cancer and the chances of developing these. Often a genetic counselor or geneticist conducts a full pedigree construction to estimate the risk. This pre-test interview is also conducted to discuss potential uncertainties, informed consent, and future preventative options. An inability to process information secondary to active psychiatric or cognitive impairment could interfere with informed consent and this should be taken into consideration by the multidisciplinary team (Patenaude, 2005). Serious depression or anxiety may mean delaying decisions about testing until consultation with mental healthcare providers (Hirschberg et al., 2015). At this stage, during the pre-test interview, the genetic counselor can identify if any individuals may experience the genetic testing procedure as stressful and can program a specific psychological intervention, if necessary. (C) *Testing*: If there is suspicion of a possible inherited risk then a genetic test, via a blood sample, is carried out and users wait from weeks to several months for the results. (D) *Post-test stage*: the multidisciplinary team then communicates the test results to the individual and helps them understand what these results mean and their implications are discussed. The test results can either be: positive, negative, or inconclusive. A positive test result indicates that the individual has a mutation that increases their chances of developing cancer. A negative test result can reassure a woman that there is no BRCA1/2 mutation, but this does not rule out other mutations in less common genes. An inconclusive test result indicates that there are variants of unknown significance and the individual may be advised to undergo further testing.

Despite heterogeneity in distress levels of subjects undergoing genetic testing, distress rates appear to be quite low, around 6–23% (Coyne et al., 2000; Hirschberg et al., 2015). However, the overload of information received during the genetic counseling sessions may be daunting for some patients. Relevant psychological issues have been raised regarding HBOC and genetic testing. How a person reacts emotionally to this new situation can affect his/her quality of life and can interfere in some delicate family dynamics. Genetic testing can bring about an awareness of a state of increased health risk with possible fears about developing cancer attached to this and perhaps feelings of anger (toward relatives who have transmitted the disease) or guilt (about potentially transmitting the mutation to one's offspring) too.

It is therefore vital that prior to the genetic test, the patient be evaluated by a counselor in terms of decision-making abilities and psychological adaptation. Psychological issues may arise even before testing takes place, for example, women with a higher perceived risk of cancer are more likely to be interested in genetic counseling (Bowen et al., 1999). Furthermore, many women regularly overestimate their risk for developing HBOC and the effectiveness of the screening procedures (Black et al., 1995). There are factors that may influence risk perception including: a family history of cancer, beliefs about cancer and its risks, loss of a family member, and a heightened identification with a family member who has or had cancer (Kash and Lerman, 1998). Thus, a well-balanced and correct presentation of the information regarding cancer risk and screening effectiveness may aid women during decision-making in the pre-test stage (Di Mattei et al., 2017). In previous studies on Huntington's disease genetic testing, psychological reactions to the testing procedure were predicted by a client's health status before the genetic test, rather than by the genetic test result itself (Tibben et al., 1993; Decruyenaere et al., 1996). The fact that test results rarely predict emotional outcomes may come as a surprise. One reason for this can be sought by examining psychological constructs which may influence emotional consequences of testing (Nordin et al., 2002), such as quality of life, psychological symptomatology, and coping mechanisms.

The relevance of coping for understanding psychological well-being among cancer patients has been shown in several studies (Ferrero et al., 1994; Nordin and Glimelius, 1998). “Coping” is a term used in psychology to define the process of adaptation of a person to a stressful situation via cognitive and behavioral efforts (Folkman and Lazarus, 1980). Theories of coping postulate that a person's reaction to a stressful situation is moderated by the ability to handle the threat and the resulting reactions. Having to undergo a genetic test is considered to be a stressful situation as the consequences of a positive outcome can be life-altering, but at the same time choosing to undergo the test can be a behavioral response through which individuals cope with the knowledge of being at a higher risk for developing cancer. Coping style has also been identified as a useful predictor of future distress during the genetic counseling process (Broadstock et al., 2000; Nordin et al., 2002) and a potential target for psychological intervention. For instance, passive coping has been associated with higher psychological distress and an unwillingness to act in decisions regarding testing (Pieterse et al., 2007; Shiloh et al., 2008). Furthermore, avoidant coping has been shown to be associated with distress over time (Dougall et al., 2009). Additionally, Di Mattei et al. (2015) showed that individuals who use avoidance coping strategies, are more vulnerable to psychological distress compared to those who use problem-oriented ones, as it seems they are inclined to deny the mutation risk. Denial and mental detachment of the problem are related to an increase of psycho-emotional distress, and constitute a psychological vulnerability factor as they predict an increase in depressive, anxious, and somatization symptoms. In other studies, coping mechanisms (Nordin et al., 2002) and pre-test emotional state have been shown to be strong predictors of distress more than 1 month after the genetic test (Broadstock et al., 2000). This is why we chose to examine pre-test distress as an indicator of possible future vulnerability to distress. In summary, examining pre-test psychological constructs can provide a better picture of how a patient can adapt and react during the genetic testing process.

The psychological impact of test results in the post-test stage has been studied extensively (Pasacreta, 2003; van Oostrom et al., 2003; van Dijk et al., 2008; Hirschberg et al., 2015) yet few studies have examined pre-test distress in cancer genetic testing in particular. The objective of the current study is to examine the levels of pre-test distress, quality of life, and active coping mechanisms in a sample of Italian women during the pre-test stage, considering that pre-test distress can be an indicator of post-test distress (Broadstock et al., 2000; Nordin et al., 2002). In detail, we evaluated differences in these psychological constructs in groups of participants that were defined based on their health status prior to genetic counseling. Once pre-test distress levels have been identified, if any, health professionals can counsel women adequately helping them to manage risks and benefits associated with testing. As the demand for genetic testing is increasing steadily, and the HBOC risk-reducing techniques have received unprecedented publicity (see the Angelina Jolie effect in Evans et al., 2014) cancer risk-assessment centers are being attended by greater numbers of individuals, currently without cancer, who are considering cancer genetic testing. Therefore, we must be prepared to meet the mental health needs of this new population and work together in identifying, counseling, testing, and managing possible patients with BRCA mutations as early on in the process as possible.

## Materials and methods

### Participants

This study included 181 female participants who accessed the Cancer Genetic Testing Service by the Medical Oncology, Breast Surgery, and Gynecology Units in a Hospital in the North of Italy between January 2012 and December 2016. The participants were subdivided into three groups on the basis of their health status: patients currently being treated for cancer, or Affected patients (*n* = 74), patients with a history of cancer, hereinafter called Ex-patients (*n* = 69) and patients with no cancer diagnosis, Unaffected participants (*n* = 38). Eligibility criteria included: (1) being at least 18 years old; (2) being able to read and understand Italian; and (3) agreed to voluntarily participate in the research. No women with severe psychiatric or neurological disorders were excluded from the study as there were none that presented these symptoms. Furthermore, no participants refused to take part in the study and all women satisfied our inclusion criteria. Informed consent was obtained from all individual participants included in the study. The study was reviewed and approved by the Medical Ethical Committee of the IRCCS San Raffaele Hospital.

### Instrumentation

Questionnaires were administered to patients during their routine sessions with a counseling psychologist in the pre-test phase, prior to their meeting with a geneticist. The first half of the session was dedicated to socio-demographic and medical history data collection. In the second half of the session the following questionnaires were administered: the Symptom Checklist-90-Revised (SCL-90), the Coping Orientation to Problems Experienced questionnaire (COPE), and the European Organization for Research and Treatment of Cancer Quality of Life Questionnaire (EORTC QLQ-C30).

The *socio-demographic data* collected included questions regarding age, marital status, and presence of children. Regarding the medical information, the following were investigated: presence of a cancer diagnosis (breast/ovarian, and previous/current), treatments that were carried out, and the pedigree relationship to any relatives that may have been diagnosed with cancer. Lastly, we also asked patients if they had ever undergone any previous psychological or psychiatric treatments, or taken any psychopharmacological drugs.

Subsequently, patients completed the following standardized questionnaires:

1. The *SCL-90-R* (Derogatis, 1994): this questionnaire evaluates a broad range of psychological problems and psychopathological symptoms. It is useful as an initial evaluation of participants at intake as an objective instrument of symptom assessment. It is composed of 90 items and the test measures 9 primary symptom dimensions: Somatization, Obsessive-Compulsive, Interpersonal Sensitivity, Depression, Anxiety, Hostility, Phobic Anxiety, Paranoid Ideation, and Psychoticism. The test is constructed to provide a summary of these symptoms and their intensity at a specific time point. Furthermore, the test provides Global Indices, which can be used as an overview of the test and these are further divided into the Global Severity Index (GSI), the Positive Symptom Distress Index (PSDI), and the Positive Symptom Total (PST). These examine overall psychological distress, intensity of symptoms, and the number of self-reported symptoms, respectively. When completing the self-administered questionnaire, the participant is asked to state on a 5-point Likert scale ranging from 1 = not at all, to 5 = extremely, whether they have experienced a series of problems or complaints during the past week. Scores > = 1 are considered clinically significant. The validity of the Italian version of the questionnaire was analyzed by Sarno et al. (2011) and these authors showed that the scale has a good internal consistency with a Cronbach's alpha that was always above 0.70 for all the scales. In the present study, we considered only 4 dimensions (Depression; Anxiety; Somatization; Hostility) and the Global Severity Index (GSI); these can be considered the best indicators of psychological distress in the context of genetic counseling.
2. *EORTC QLQ-C30 Version 3.0* (Aaronson et al., 1993): is a self-administered instrument composed of 30 items and includes a global health and quality of life scale, five functional subscales evaluating physical, role, emotional, cognitive, and social functioning and three symptom subscales evaluating nausea and vomiting, pain, and fatigue. Six single items assess financial difficulties and symptoms that are frequently reported by cancer patients (dyspnea, insomnia, appetite loss, constipation, and diarrhea). Responses are given on a 4-point Likert Scale from 1 (not at all) to 4 (very much), with total scores ranging from 0 to 100; higher scores correspond to a better level of functioning for the functional and global scales and to a higher severity of symptoms for the symptom scales. The psychometric properties of the questionnaire were tested and it was found to possess the required standards of validity, reliability, and sensitivity (Aaronson et al., 1993; Osoba et al., 1994; Kaasa et al., 1995).
3. *COPE* (Carver et al., 1989): this inventory was designed to assess people's coping responses (dysfunctional and functional) to stress or difficult situations. We used the Italian version of the questionnaire, called the *COPE-NVI* (Sica et al., 2008), which is composed of 60 items using a Likert scale ranging from 1 = I usually don't do this at all, to 4 = I usually do this a lot. After a factor analysis, the items of the Italian version were divided into 5 dimensions (we used standardized scores): Social support, Avoidance strategies, Positive attitude, Problem solving, and Turning to religion. Statistical analysis of the Italian version has revealed good internal consistency with Cronbach's alpha scores ranging from 0.70 to 0.91 (Sica et al., 2008).

### Data analysis

Descriptive statistics for continuous variables, stratified by group, have been presented as mean and standard deviation, while frequency distribution has been reported for categorical variables. To compare socio-demographic and clinical characteristics among groups, Fisher's exact test was applied.

To identify differences among Unaffected patients, Affected patients, and Ex-patients on the psychological constructs of interest and due to the non-normality of the examined psychometric scales, a Kruskal–Wallis test, the nonparametric counterpart to standard ANOVA, followed by *post-hoc* analysis (Dunn's pairwise test and Bonferroni's adjustment of *p*-values), have been applied.

All the analyses were performed using R statistical software (R. Core Team, 2016) and the significance threshold was set at 0.05. The *PMCMR* package, developed in R, was used to implement the Kruskal–Wallis test and *post-hoc* analysis for pairwise multiple comparisons (Pohlert, 2014).

## Results

The mean age of the study participants was 48.54 years (*SD* = 11.54), ranging from 21 to 80 years. As mentioned previously, the participants were divided into 3 groups on the basis of their health status: Affected patients (40.88%; *n* = 74), “Ex-patients” (38.12% *n* = 69), and Unaffected participants (20.99%; *n* = 38). In Table 1, socio-demographic and clinical characteristics for each group are shown. As can be seen in Table 1, we found significant differences between the three groups with regard to the presence of children and the treatment type (surgery and chemotherapy).

**Table 1:** Socio-demographic and clinical characteristics for each group of participants.

	**Patients (*****n*** = **74)**		**Ex-patients (*****n*** = **69)**		**Unaffected participants (*****n*** = **38)**		***p*-value**
**Characteristic**	**Freq**.	**%**	**Freq**.	**%**	**Freq**.	**%**	
Marital status							0.19
In a relationship	62	84.93	48	72.73	29	76.32	
No relationship (Single; Divorced; Widow)	11	15.07	18	27.27	9	23.68	
Presence of children							0.02
No	11	14.86	16	23.88	15	39.47	
Yes	63	85.14	51	76.12	23	60.53	
Diagnoses							0.75
Breast cancer	56	76.71	51	75	–	–	
Gynecologic cancers	12	16.44	14	20.59	–	–	
Both	5	6.85	3	4.41	–	–	
Radiation therapy							0.05
No	46	62.16	35	52.24	–	–	
Yes, currently	6	8.11	1	1.49	–	–	
Before genetic counseling	22	29.73	31	46.27	–	–	
Chemotherapy treatment (currently or before)							<0.01
No	14	18.92	24	35.29	–	–	
Yes, currently	43	58.11	1	1.47	–	–	
Before genetic counseling	17	22.97	43	63.24	–	–	
Surgery							<0.01
No	18	24.32	5	7.35	–	–	
Yes	56	75.68	63	92.65	–	–	
Specifically							
Mastectomy	12	22.64	10	17.54	–	–	
Quadrantectomy	27	50.94	26	45.61	–	–	
Bilateral mastectomy	2	3.77	2	3.51	–	–	
Bilateral quadrantectomy	2	3.77	1	1.75	–	–	
Hysterectomy/oophorectomy	7	13.21	11	19.30	–	–	
Other	3	5.66	7	12.28	–	–	
Previous psych. int.							0.80
No	53	73.61	47	70.15	29	76.32	
Yes	19	26.39	20	29.85	9	23.68	
Psychotropic med.							0.09
No	58	79.45	54	80.60	36	94.74	
Yes	15	20.55	13	19.40	2	5.26	
Familiarity with cancer							0.56^*^
No	20	27.40	15	22.06	0	0	
Yes	53	72.60	53	77.94	38	100	

* *Patients vs. Ex.patients*.

Descriptive statistics of variables of interest, along with group comparisons, are presented in Tables 2–4.

**Table 2:** SCL-90-R scores for each group of participants.

		**M (SD)**	**Med.(Q1-Q3)**	***p*-value KS**	**Adjusted *p*-values pairwise comparisons**
Global severity index	Patients	0.48 (0.45)	0.4 (0.2–0.56)	<0.01	1^a^, <0.01^b^, <0.01^c^
	Ex-patients	0.41 (0.29)	0.4 (0.19–0.53)		
	P. with no cancer	0.22 (0.17)	0.18 (0.1–0.28)		
Somatization	Patients	0.82 (0.72)	0.67 (0.33–1)	<0.01	0.30^a^, <0.01^b^
	Ex-patients	0.59 (0.44)	0.5 (0.25–0.75)		<0.01^c^
	P. with no cancer	0.36 (0.4)	0.29 (0.1–0.42)		
Depression	Patients	0.65 (0.69)	0.38 (0.15-0.9)	<0.01	1^a^, <0.01^b^, 0.01^c^
	Ex-patients	0.54 (0.52)	0.38 (0.15–0.77)		
	P. with no cancer	0.26 (0.26)	0.15 (0.1–0.31)		
Anxiety	Patients	0.5 (0.62)	0.4 (0.12–0.57)	0.02	1^a^, 0.07^b^, 0.02^c^
	Ex-patients	0.45 (0.37)	0.4 (0.2–0.6)		
	P. with no cancer	0.27 (0.27)	0.2 (0.1–0.4)		
Hostility	Patients	0.3 (0.44)	0.17 (0–0.33)	0.11	
	Ex-patients	0.28 (0.31)	0.17 (0–0.5)		
	P. with no cancer	0.18 (0.3)	0.17 (0–0.17)		

a Patients vs. Ex-patients,

b Patients vs. P. with no cancer,

c *Ex-patients vs. P. with no cancer*.

**Table 3:** EORTC QLQ-C30 scores for each group of participants.

		**M (SD)**	**Med.(Q1-Q3)**	***p*-value KS**	**Adjusted *p*-values pairwise comparisons**
Global health scale	Affected	67.34 (21.13)	66.67 (52.08–83.33)	<0.01	0.41^a^
	Ex-patients	71.57 (21.81)	75 (66.67–83.33)		<0.01^b^, 0.13^c^
	Unaffected	80.13 (15.23)	83.33 (75–91.67)		
Physical functioning	Affected	83.65 (19.69)	90 (75–100)	<0.01	0.42^a^
	Ex-patients	89.76 (12.24)	93.33 (86.67–100)		<0.01^b^, <0.01^c^
	Unaffected	97.54 (4.76)	100 (95–100)		
Role functioning	Affected	78.83 (29.23)	100 (66.67–100)	<0.01	1^a^
	Ex-patients	80.02 (24.91)	83.33 (66.67–100)		<0.01^b^, <0.01^c^
	Unaffected	96.93 (8.54)	100 (100–100)		
Emotional functioning	Affected	73.65 (25.02)	75 (66.67–91.67)	0.12	
	Ex-patients	77.17 (20.99)	83.33 (66.67–91.67)		
	Unaffected	83.77 (16.2)	87.5 (75–100)		
Cognitive functioning	Affected	83.11 (19.99)	83.33 (66.67–100)	0.02	1^a^
	Ex-patients	81.55 (21.73)	83.33 (66.67–100)		0.05^b^, 0.02^c^
	Unaffected	91.23 (16.77)	100 (87.5–100)		
Social functioning	Affected	79.95 (28.8)	100 (66.67–100)	<0.01	1^a^
	Ex-patients	83.74 (24.48)	100 (66.67–100)		<0.01^b^, <0.01^c^
	Unaffected	94.88 (17.15)	100 (100–100)		

a Affected vs. Ex-patients,

b Affected vs. Unaffected,

c *Ex-patients vs. Unaffected*.

**Table 4:** COPE-NVI scores for each group of participants.

		**M (SD)**	**Med.(Q1-Q3)**	***p*-value KS**	**Adjusted *i-*values pairwise comparisons**
Social support	Affected	51.13 (22.01)	50 (36.11–68.75)	0.02	1^a^
	Ex-patients	49.28 (21.38)	47.22 (33.33–63.89)		0.05^b^, 0.02^c^
	Unaffected	60.89 (17.81)	63.89 (53.47–69.44)		
Avoidance strategies	Affected	14.3 (10.65)	13.54 (6.25–20.83)	0.28	
	Ex-patients	14.91 (10.54)	12.5 (6.25–22.92)		
	Unaffected	11.57 (8.93)	8.33 (4.17–16.15)		
Positive attitude	Affected	63.37 (15.5)	66.67 (55.56–72.22)	0.66	
	Ex-patients	64.33 (16.09)	63.89 (52.78–75)		
	Unaffected	62.21 (14.07)	59.72 (52.78–71.53)		
Problem solving	Affected	54.47 (18.18)	54.17 (42.36–66.67)	0.59	
	Ex-patients	53.86 (15.99)	52.78 (44.44–63.89)		
	Unaffected	56.73 (16.13)	55.56 (50.69–65.97)		
Turning to religion	Affected	63.46 (23.84)	60.42 (45.83–83.33)	0.17	
	Ex-patients	61.11 (22.76)	58.33 (45.83–75)		
	Unaffected	54.93 (21.83)	50 (45.83–65.63)		

a Affected vs. Ex-patients,

b Affected vs. Unaffected,

c *Ex-patients vs. Unaffected*.

Focusing on the SCL-90-R, no group reported any scores of clinical relevance. Based on the Kruskal–Wallis test, groups of participants were significantly different in terms of GSI (*p* < 0.0001), Anxiety (*p* = 0.0221), Somatization (*p* < 0.0001), and Depression (*p* = 0.0028).

In particular, a significant difference emerged between Ex-patients and Unaffected participants in the mean scores of the GSI (*p* = 0.0012), Anxiety (*p* = 0.023), Somatization (*p* = 0.0023), and Depression (*p* = 0.0146). For both GSI and Somatization, the group of Affected patients significantly differed from those with no cancer diagnosis (*p* < 0.0001). Moreover, their scores significantly differed on the Depression scale (*p* = 0.0027).

Relatively good levels of health-related quality of life were reported by all groups in all functional domains. Overall, groups were significantly different in terms of their Global health (*p* = 0.0045), Physical (*p* < 0.0001), Cognitive (*p* = 0.0156), Social (*p* = 0.0012), and Role Functioning (*p* = 0.0001). The scores of the Global health scale, Physical, Cognitive, Social, and Role Functioning are significantly different between the Affected patients and the Unaffected participants (*p* = 0.0031; *p* < 0.0001, *p* = 0.0487; *p* = 0.0011; *p* = 0.0005, respectively). The healthy group also differed from the group of Ex-patients regarding Physical, Cognitive, Social, and Role Functioning (*p* = 0.0015; *p* = 0.0164; *p* = 0.0095; *p* = 0.0001, respectively).

The only coping strategy that revealed statistically significant differences among the subgroups was the social support coping mechanism (*p* = 0.0171). In detail, there was a significant difference between patients without cancer and those with cancer, both in the present or in the past (*p* = 0.048; *p* = 0.0191, respectively). We did not find any significant differences between the Affected patients and Ex-patients on the considered constructs.

## Discussion

Presently, the demand for genetic testing is increasing steadily, both for patients and healthy individuals. Therefore, it becomes fundamental to address psychological issues even before testing takes place, so as to identify the presence of pre-test distress and its potential relationship with that of post-test distress (Broadstock et al., 2000; Nordin et al., 2002). In their systematic literature review, Broadstock et al. (2000) found that the two variables which were most often used in studies to predict emotional consequences of genetic testing were the genetic test result and the pre-test emotional state, however, the test result was rarely predictive of distress more than 1 month after testing, whereas pre-test emotional state was a much stronger predictor. We therefore aimed to identify the level of pre-test distress in our sample of women and see if there were any differences with regards to different psychological constructs between the three subgroups which were divided on the basis of participant health status during the pre-test phase.

The literature regarding cancer genetic counseling for BRCA1/2 genes has consistently found that about 10% of patients who undertake the counseling procedure develop clinically significant symptoms of psychological distress, both in the long and short term, and with a positive test result (Halbert et al., 2011; Graves et al., 2012). In this small number of women, this type of psychological distress can negatively influence individual health, for example, prevention procedures may be neglected; the management of both personal cancer risk and that of relatives could then become more complex.

As the level of distress shown in patients in the pre-test phase can be considered a significant predictor of post-test psychological distress, the present study aimed to evaluate clinical distress in the pre-test phase by focusing on psychological symptoms, quality of life, and coping strategies. Although previous studies observed that carriers of BRCA1/2 genes seemed more vulnerable to manifesting distress at the end of the genetic counseling process (Graves et al., 2012), to our knowledge, no study has examined whether different subgroups of women present different vulnerabilities based on their health status during the pre-test phase in a cancer genetic counseling context. This vulnerability could have an impact on the management of the test results and it is therefore useful to identify this vulnerability as early on in the process as possible. In the present study, we chose to consider three subcategories of patients: Affected patients, Unaffected participants, and Ex-patients.

Regarding psychological distress, measured via the Global Severity Index, no group of patients presented levels that could be considered clinically significant. This result confirms what has been found previously in the literature (Halbert et al., 2011; Graves et al., 2012; Hirschberg et al., 2015). The comparison between the three groups of participants showed that Affected patients and Ex-patients presented a significantly different number of psycho-emotional symptoms compared to the Unaffected participants. Specifically, both groups of patients showed more depressive symptomatology: this result is in line with the previous literature, in that patients in the active treatment phase report higher levels of distress (Meiser, 2005). With respect to Ex-patients, the higher level of distress could be due to the physical and psychological consequences of previous treatments and their worries about the future, together with the fear of possible disease relapse. Both groups could also erroneously overestimate the risk of being a carrier of BRCA1/2 (Black et al., 1995; Bowen et al., 1999), compared to Unaffected participants.

The data regarding anxiety symptoms also reflects what has been found previously in the literature: there is a significant difference between Ex-patients and Unaffected participants. This result could be explained by the state of uncertainty that Ex-patients find themselves in, coupled with the fear of a possible relapse; a positive test result may in turn result in disease recurrence. The presence of a genetic mutation could also force Ex-patients to newly reconsider their life choices, evoking once more the disease condition, which was experienced previously during the active phase of the disease. This means that Ex-patients must also confront other implications, including prognostic consequences and an increased future risk of disease (Suchocka-Capuano and Bungener, 2010). In line with our expectations, the results of the SCL-90-R questionnaire revealed higher somatization symptom scores in Ex-patients and Affected patients compared to Unaffected participants; this result could be explained by the perception of a more precarious health status in patients who have or have had an oncological illness or the presence of side effects in their medical treatments.

The analyses concerning quality of life showed that the sample of women in this study maintained an adequate state of well-being and functionality when it came to the aspects measured by the EORTC QLQ-30. The results revealed that participants could keep active as well as cultivate social relationships. The general health status, even though reasonable, was significantly lower in Affected patients compared to those without the disease; this result may be due primarily to the physical symptoms of the disease itself, and secondly to the side effects of the treatments associated to cancer.

Another interesting finding is the significant difference between Ex-patients and Unaffected individuals with regard to physical functioning. This difference could be explained by the fact that in women who have already been through the disease process, side effects may persist even after treatment is over, whereas Unaffected individuals will not have confronted such physical issues. Thus, Unaffected participants generally start from a higher baseline of physical functioning compared to patients (Muzzatti et al., 2015).

Furthermore, the findings that role functioning, cognitive functioning, and social functioning are significantly different between Unaffected participants and the other two subgroups are also in agreement with the previous literature (Klein et al., 2011). We hypothesize that the main reason for this reduced functionality is the negative impact of a cancer diagnosis and the treatments themselves on the ability of patients to carry out their normal daily activities during the active phase of the disease and on the possibility of regaining their previous daily functionality and social routine after treatments have terminated. This is even more evident in women who have been subjected to very invasive treatments. The inability to fully repossess one's previous role could be perceived as a very difficult objective to obtain in the case of a positive test result.

The cancer diagnosis and its treatments may not only alter the physical and mental condition of a patient but could also have a negative impact on the patient's social relations, thus reducing not only the possibility to continue one's social life but also the wish to do so. Moreover, it is interesting to note that the two groups of patients do not present a significant difference in all of the dimensions analyzed in the present study; this finding could be down to the fact that both groups experience side effects of cancer treatments (both in the long and short term) and live with a history of illness.

The literature concerning coping mechanisms shows that people favor either a problem-focused approach or an emotional-focused one (Bennet and Soulsby, 2012). The findings from this study reveal that there is not a clear preference within our sample, and that all the different coping mechanisms used are in some way called upon to face the cancer genetic counseling process. Avoidance was the coping mechanism that was used less frequently within the three subgroups. This coping mechanism determines a psychological distancing and a disinvestment in resources on behalf of the participant when confronting the problem at hand; undertaking the genetic counseling process is in itself an indicator that these participants did not avoid the issue, in fact, they focused their attention and used their resources to understand their health status and try to prevent or cure their condition.

The only coping mechanism that seems to be involved in discriminating among group participants is the social support coping mechanism, with Unaffected individuals showing significantly different scores on this construct when compared to the other two subgroups of patients. This finding can be interpreted considering what we have already stated above: people who must face an illness have more limited social functioning and for this reason they may rely less on their social network for support.

### Study limitations and research recommendations

Certain limitations of the study must also be considered. Firstly, within the Ex-patient subgroup are both those who had recently concluded their treatments and thus still experiencing side effects, and those who had concluded their treatments years ago, and were therefore probably over the worst of their treatment side effects. Perhaps in the future we can further subdivide this group to differentiate between these two categories of Ex-patients. It would be interesting to consider different illness severities within the Affected and Ex-patient groups, especially with respect to the staging and the presence of metastases; women with metastatic breast cancer in fact show a poorer quality of life (Yen et al., 2006). Furthermore, we did not take into consideration possible variables that were not connected to the oncological condition of the patient, for instance, other illnesses or stressful life events, which may have impacted on the psychological and physical condition of the participants during testing. In the future, we will investigate this aspect and also the motivation as to why these women decided to undergo the genetic testing procedure. In this study, we also did not examine certain socio-demographic factors that could in fact be interesting on a clinical level, for example, age, the presence of children, work status, or the presence of social support. For example, younger participants could have more resources and may be more likely to make up the Unaffected participant group; mothers could experience more distress for fear of having transmitted the mutation to their offspring. It would also be opportune to examine what cultural, social, religious, and family resources patients have in future studies. These data could impact the psychological symptomatology, especially regarding distress and the functioning scales. Lastly, this study was cross-sectional and thus limited the measurements to only the pre-test phase. In the future, we foresee conducting a longitudinal study and comparing the pre-test and post-test results, and perhaps looking at the long-term adaptation of patients undertaking a cancer genetic counseling process.

### Practice implications

The present study can be located in a relatively new research field in which the objective is to examine the psychological adaptation of participants to the cancer genetic counseling process. The results from this study confirm what has already been found in the literature, that is, the genetic counseling procedure determines a scant number of clinically significant psychological cases. However, certain groups of participants were identified as being more vulnerable than others as in the pre-test phase they reported higher levels of distress and lower quality of life scores. Therefore, not all individuals who access the cancer genetic counseling process are the same. In our study the participants with these characteristics were predominantly in the Ex-patient and Affected patient groups, thus these individuals may be more at risk of distress during the cancer genetic counseling process. In light of these considerations, we suggest that clinicians, oncologists, geneticists, and counselors pay particular attention to these subgroups of patients during the pre-test phase. These patients should be allowed to voice their worries, perplexities, and expectations with regard to the genetic counseling procedure and results. A psychoeducational approach could be used to inform patients with respect to the counseling process and the meaning of the results; it is possible that a greater awareness and knowledge of the process could allow patients to use more adaptive coping strategies and thus adapt better to the counseling procedure.

## Ethics statement

All procedures performed in studies involving human participants were in accordance with the ethical standards of the institutional research committee (San Raffaele Hospital) and with the 1964 Helsinki declaration and its later amendments or comparable ethical standards. All subjects gave written informed consent in accordance with the Declaration of Helsinki.

## Author contributions

VD was responsible for the conception, interpretation and design of the research project and approving the final copy. LC was responsible for drafting the work, revising it critically, drafting the study design and data acquisition. MB and RB were responsible for drafting the work, data collection, revising the manuscript and conducting research of the intellectual content. CB and FC were responsible for statistical analyses and data interpretation. ER and MZ were the oncologists responsible with organizing the project and helping with data collection and proof checking of the manuscript. MC, LS, and OG were responsible for the conception and design of the research project, critical analysis and approving the final copy. All authors are thus accountable for all aspects of the work, also with respect to the integrity and accuracy of it.

### Conflict of interest statement

The authors declare that the research was conducted in the absence of any commercial or financial relationships that could be construed as a potential conflict of interest.

## Footnotes

^1^Associazione Italiana di Oncologia Medica. *L'incidenza dei Tumori in Italia (The Incidence of Tumours in Italy)*. Available online at: http://www.registri-tumori.it/PDF/AIOM2016/I_numeri_del_cancro_2016.pdf
